# Challenges in the design and analysis of surgical trials

**DOI:** 10.1186/1745-6215-14-S1-P31

**Published:** 2013-11-29

**Authors:** Ines Rombach, David Beard, Andrew Carr, Jonathan Rees, Cushla Cooper, Naomi Cummings

**Affiliations:** 1 University of Oxford, Oxford, UK

## 

The development of surgical techniques differs greatly from that of new drugs. Surgical procedures often become widely used and accepted without any evidence from RCTs to show their superiority or non-inferiority to alternative medical treatments.

This phenomenon, together with varying waiting-times for surgeries and patient expectations can place a number of constraints on the design of surgical trials, which impact greatly on the planned analysis of such studies. Often, these factors are working against regulators' increasing awareness of the need to thoroughly assess the effectiveness of surgical procedure.

This presentation discusses some of these challenges and demonstrates how these were approached in CSAW, a trial assessing the effectiveness of sub-acromial decompression surgery for shoulder pain.

There are conflicting demands on trial design and planned analysis. The most evident of these is that clinical researchers are interested in the effect of the treatment from the intervention (i.e. the surgery), whereas conventional statistical techniques place outcome assessments at specific time-points from randomisation. Differences between both strategies are particularly pronounced in trials measuring short-term outcomes or comparing surgical treatments with potentially long waiting-times and non-surgical interventions. There is tension between the need to accurately reflect the effects of a major intervention compared to need to account for any other effects, including the passage of time. Both conflicting viewpoints have advantages and limitations. This paper considers the decision-making process leading to a trial design and analysis plan that considers a compromise between appropriate trials methodology, patient concerns and expectations, as well as surgeon objectives.

